# Supplementary material for: Validation of suitable genes for normalization of diurnal gene expression studies in Chenopodium quinoa
Source: PLoS One. 2021 Mar 11;16(3):e0233821. doi: 10.1371/journal.pone.0233821 (PMC7951847; doi:10.1371/journal.pone.0233821)
Supplement: S1 Table — (PDF) [file pone.0233821.s001.pdf]

S1 Table.

Zeitgeber point	Biological replicate	A_{260}/A_{280} ratio	A_{260}/A_{230} ratio
ZT-0	1	2.14	2.38
	2	2.16	2.46
	3	2.14	2.40
ZT-4	1	2.15	2.34
	2	2.12	2.30
	3	2.16	2.33
ZT-8	1	2.14	1.89
	2	2.15	1.87
	3	2.14	2.08
ZT-12	1	2.19	2.36
	2	2.18	2.44
	3	2.16	2.43
ZT-16	1	2.17	2.42
	2	2.18	2.45
	3	2.18	2.40
ZT-20	1	2.15	1.78
	2	2.10	1.71
	3	2.14	2.33
ZT-24	1	2.21	2.27
	2	2.17	2.43
	3	2.18	2.40
