# Supplementary material for: Validation of suitable genes for normalization of diurnal gene expression studies in Chenopodium quinoa
Source: PLoS One. 2021 Mar 11;16(3):e0233821. doi: 10.1371/journal.pone.0233821 (PMC7951847; doi:10.1371/journal.pone.0233821)
Supplement: S2 Table — Selected genes were stably expressed at least in two out of four stress conditions in soil- and hydroponic-grown plants. (PDF) [file pone.0233821.s002.pdf]

S2 Table.

Shoot tissue					
Gene ID	Gene abbreviation	FPKM[1]			
		Low phospate	Salt	Drought	Heat
AUR62005573	*ACT*	17.55 ± 6.61 [a]	8.72 ± 0.96 [a]	7.47 ± 2.57 [a]	6.96 ± 0.07 [a]
AUR62005167	*GAPDH*	0.72 ± 0.51 [a]	0.33 ± 0.13 [a]	0.29 ± 0.21 [a]	0.32 ± 0.05 [a]
AUR62030674	*RAN-3*	253.36 ± 39.22 [a]	339.90 ± 22.29 [b]	272.08 ± 12.29 [a,b]	334.70 ± 26.97 [a,b]
AUR62002238	*IDH-A*	22.90 ± 7.54 [b]	12.58 ± 0.79 [a,b]	14.98 ± 2.64 [a,b]	8.37 ± 1.30 [a]
AUR62037769	*IDH-B*	4.64 ± 0.44 [a]	5.22 ± 0.93 [a]	5.76 ± 0.53 [a]	6.03 ± 1.21 [a]
AUR62034430	*PTB*	21.35 ± 4.45 [a]	19.83 ± 0.40 [a]	16.62 ± 0.76 [a]	16.66 ± 1.11 [a]
AUR62007961	*TUB*	238.74 ± 114.89 [a]	331.87 ± 30.47 [a]	196.15 ± 27.11 [a]	300.68 ± 21.49 [a]
AUR62015654	*UBQ*	1,171.62 ± 65.90 [b]	871.21 ± 60.65 [a]	1,245.93 ± 74.36 [b]	1,100.60 ± 138.49 [a,b]
**Root tissue**					
**Gene ID**	**Gene abbreviation**	**FPKM[1]**			
		**Low phospate**	**Salt**	**Drought**	**Heat**
AUR62005573	*ACT*	19.18 ± 7.05 [a]	9.74 ± 2.32 [a]	11.64 ± 0.87 [a]	14.09 ± 0.27 [a]
AUR62005167	*GAPDH*	33.93 ± 17.30 [b]	7.96 ± 0.98 [a]	4.04 ± 0.42 [a]	11.66 ± 2.30 [a,b]
AUR62030674	*RAN-3*	274.44 ± 3.36 [b]	276.42 ± 7.50 [b,c]	258.68 ± 2.02 [a]	298.96 ± 1.38 [d]
AUR62002238	*IDH-A*	8.55 ± 0.31 [a]	8.46 ± 0.52 [a]	7.82 ± 0.16 [a]	8.29 ± 1.38 [a]
AUR62037769	*IDH-B*	5.55 ± 0.56 [a]	6.30 ± 0.61 [a]	5.22 ± 0.20 [a]	6.32 ± 0.26 [a]
AUR62034430	*PTB*	15.49 ± 1.01 [a]	17.25 ± 0.54 [a]	16.49 ± 0.90 [a]	14.16 ± 2.62 [a]
AUR62007961	*TUB*	261.90 ± 50.90 [a]	360.43 ± 1.75 [a,b]	292.32 ± 22.62 [a,b]	370.32 ± 31.24 [b]
AUR62015654	*UBQ*	995.33 ± 484.06 [a]	1,022.63 ± 27.48 [a,b]	1,263.08 ± 135.35 [a,b]	1,846.38 ± 112.62 [b]

Letters show significant differences between stress conditions (Tukey's Multiple Comparison Test; α=0.05)

[1] FPKM=Fragments Per Kilobase Million
