# Supplementary material for: Validation of suitable genes for normalization of diurnal gene expression studies in Chenopodium quinoa
Source: PLoS One. 2021 Mar 11;16(3):e0233821. doi: 10.1371/journal.pone.0233821 (PMC7951847; doi:10.1371/journal.pone.0233821)
Supplement: S3 Table — (PDF) [file pone.0233821.s003.pdf]

S3 Table. Minimum Information for Publication of Quantitative Real-Time PCR Experiments (MIQE): checklist.

ITEM TO CHECK	IMPORTANCE	CHECKLIST
**EXPERIMENTAL DESIGN**		
Definition of experimental and control groups	E	yes
Number within each group	E	yes
Assay carried out by core lab or investigator's lab?	D	no
Acknowledgement of authors' contributions	D	yes
**SAMPLE**		
Description	E	yes
Volume/mass of sample processed	D	yes
Microdissection or macrodissection	E	NA
Processing procedure	E	yes
If frozen - how and how quickly?	E	yes
If fixed - with what, how quickly?	E	NA
Sample storage conditions and duration (especially for FFPE samples)	E	yes
**NUCLEIC ACID EXTRACTION**		
Procedure and/or instrumentation	E	yes
Name of kit and details of any modifications	E	yes
Source of additional reagents used	D	yes
Details of DNase or RNAse treatment	E	yes
Contamination assessment (DNA or RNA)	E	yes
Nucleic acid quantification	E	yes
Instrument and method	E	yes
Purity (A260/A280)	D	yes
Yield	D	yes
RNA integrity method/instrument	E	yes
RIN/RQI or Cq of 3' and 5' transcripts	E	by Agarose electrophoresis
Electrophoresis traces	D	yes
Inhibition testing (Cq dilutions, spike or other)	E	yes
**REVERSE TRANSCRIPTION**		
Complete reaction conditions	E	yes
Amount of RNA and reaction volume	E	yes
Priming oligonucleotide (if using GSP) and concentration	E	yes
Reverse transcriptase and concentration	E	yes
Temperature and time	E	yes
Manufacturer of reagents and catalogue numbers	D	yes
Cqs with and without RT	D*	no
Storage conditions of cDNA	D	no
**qPCR TARGET INFORMATION**		
If multiplex, efficiency and LOD of each assay.	E	NA
Sequence accession number	E	yes
Location of amplicon	D	yes
Amplicon length	E	yes
In silico specificity screen (BLAST, etc)	E	yes
Pseudogenes, retropseudogenes or other homologs?	D	no
Sequence alignment	D	yes
Secondary structure analysis of amplicon	D	no
Location of each primer by exon or intron (if applicable)	E	yes
What splice variants are targeted?	E	yes
**qPCR OLIGONUCLEOTIDES**		
Primer sequences	E	yes
RTPrimerDB Identification Number	D	no
Probe sequences	D**	no
Location and identity of any modifications	E	yes
Manufacturer of oligonucleotides	D	yes
Purification method	D	no
**qPCR PROTOCOL**		
Complete reaction conditions	E	yes
Reaction volume and amount of cDNA/DNA	E	yes
Primer, (probe), Mg++ and dNTP concentrations	E	yes
Polymerase identity and concentration	E	yes
Buffer/kit identity and manufacturer	E	yes
Exact chemical constitution of the buffer	D	no
Additives (SYBR Green I, DMSO, etc.)	E	yes
Manufacturer of plates/tubes and catalog number	D	no
Complete thermocycling parameters	E	yes
Reaction setup (manual/robotic)	D	no
Manufacturer of qPCR instrument	E	yes
**qPCR VALIDATION**		
Evidence of optimisation (from gradients)	D	no
Specificity (gel, sequence, melt, or digest)	E	yes
For SYBR Green I, Cq of the NTC	E	yes
Standard curves with slope and y-intercept	E	yes
PCR efficiency calculated from slope	E	yes
Confidence interval for PCR efficiency or standard error	D	no
r2 of standard curve	E	yes
Linear dynamic range	E	yes
Cq variation at lower limit	E	NA (fixed baseline threshold)
Confidence intervals throughout range	D	no
Evidence for limit of detection	E	yes
If multiplex, efficiency and LOD of each assay.	E	NA
**DATA ANALYSIS**		
qPCR analysis program (source, version)	E	yes
Cq method determination	E	yes
Outlier identification and disposition	E	yes
Results of NTCs	E	yes
Justification of number and choice of reference genes	E	yes
Description of normalisation method	E	yes
Number and concordance of biological replicates	D	yes
Number and stage (RT or qPCR) of technical replicates	E	yes
Repeatability (intra-assay variation)	E	yes
Reproducibility (inter-assay variation, %CV)	D	no
Power analysis	D	yes
Statistical methods for result significance	E	yes
Software (source, version)	E	NA
Cq or raw data submission using RDML	D	upon request

**E=**	essential informaiton
**D=**	desirable informaiton
**Submitted=**	yes
**Not provided=**	no
**Not aplicable=**	NA

*: Assessing the absence of DNA using a no RT assay is essential when first extracting RNA. Once the sample has been validated as RDNA-free, inclusion of a no-RT control is desirable, but no longer essential.

**: Disclosure of the probe sequence is highly desirable and strongly encouraged. However, since not all commercial pre-designed assay vendors provide this information, it cannot be an essential requirement. Use of such assays is advised against.
