# Supplementary figures and images for: Validation of suitable genes for normalization of diurnal gene expression studies in Chenopodium quinoa
Source: PLoS One. 2021 Mar 11;16(3):e0233821. doi: 10.1371/journal.pone.0233821 (PMC7951847; doi:10.1371/journal.pone.0233821)

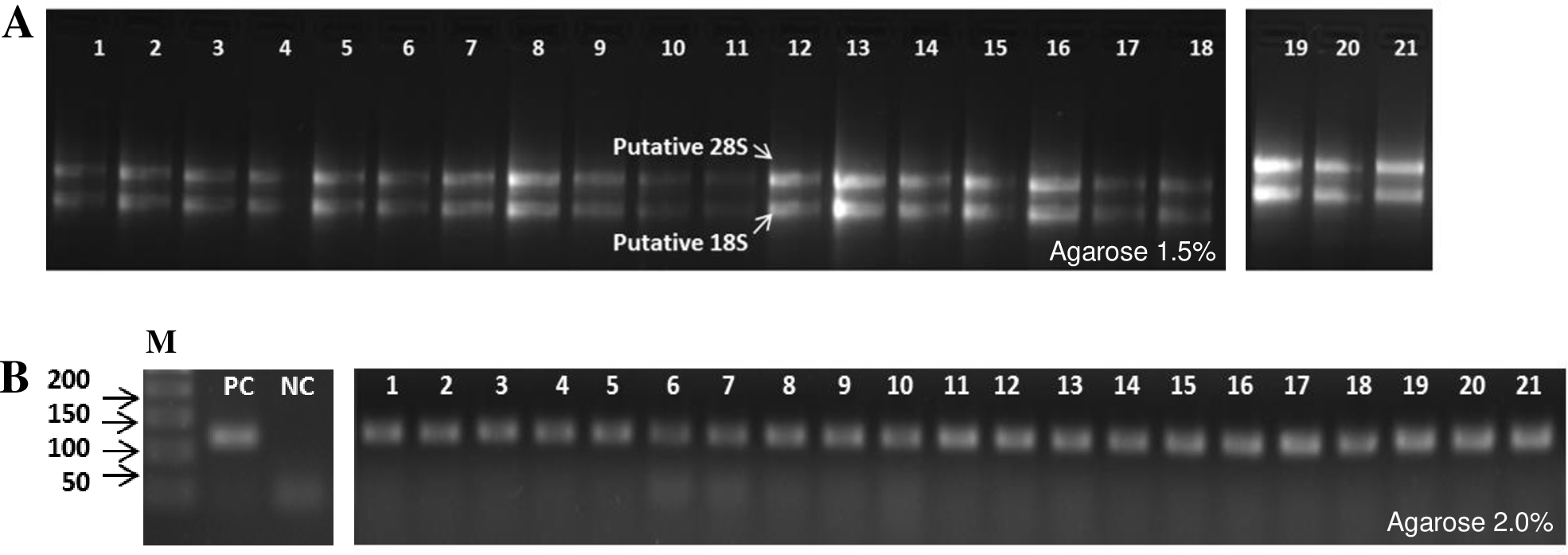

Supplement: S1 Fig — (A) 1.5% agarose gel of RNA isolated from leaf samples of the Titicaca accession; lanes 1 to 3: ZT-0, lanes 4 to 6: ZT-4, lanes 7 to 9: ZT-8, lanes 10 to 1, lanes 13 to 15: 2: ZT-12, lanes 13 to 15: ZT-16, lanes 16 to 18: ZT-20, lanes 19 to 21: ZT-24. Putative bands for 28S and 18S rRNA are indicated by arrows. Agarose gel was run for 40 min at 100 V (cropped gel). (B) 2.0% agarose gel of PCR products of cDNA synthesized from RNA leaf samples of Titicaca accession; lanes 1 to 3: ZT-0, lanes 4 to 6: ZT-4, lanes 7 to 9: ZT-8, lanes 10 to 1, lanes 13 to 15: 2: ZT-12, lanes 13 to 15: ZT-16, lanes 16 to 18: ZT-20, lanes 19 to 21: ZT-24. M: 50 bp ladder, PC: positive control, NC: water. Agarose gel was run for 30 min at 100 V (cropped gel). (TIF) [file pone.0233821.s004.tif]

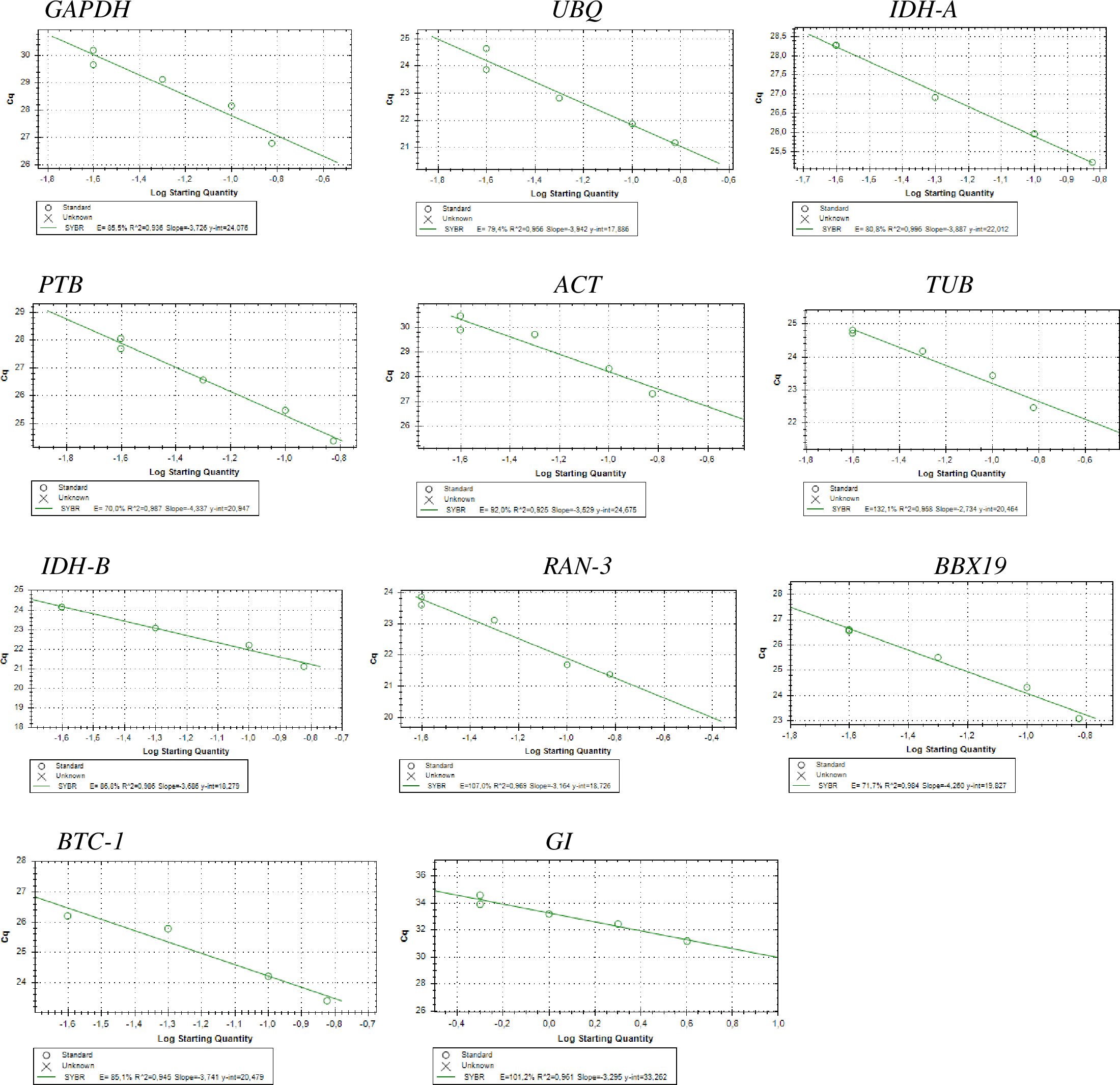

Supplement: S2 Fig — Dilution factors 1:40 (2x), 1:20, 1:10 and 3:20 were used. (TIF) [file pone.0233821.s005.tif]

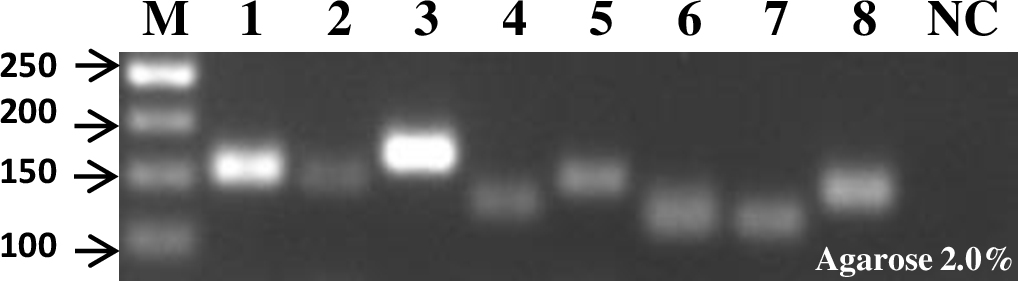

Supplement: S3 Fig — Lane 1: ACT, lane 2: GAPDH, lane 3: IDH-B, lane 4: IDH-A, lane 5: PTB, lane 6: TUB, lane 7: UBQ, lane 8: RAN-3, NC: water, M: 50 bp ladder. 2.0% agarose gel was run for 40 min at 100 V. (TIF) [file pone.0233821.s006.tif]

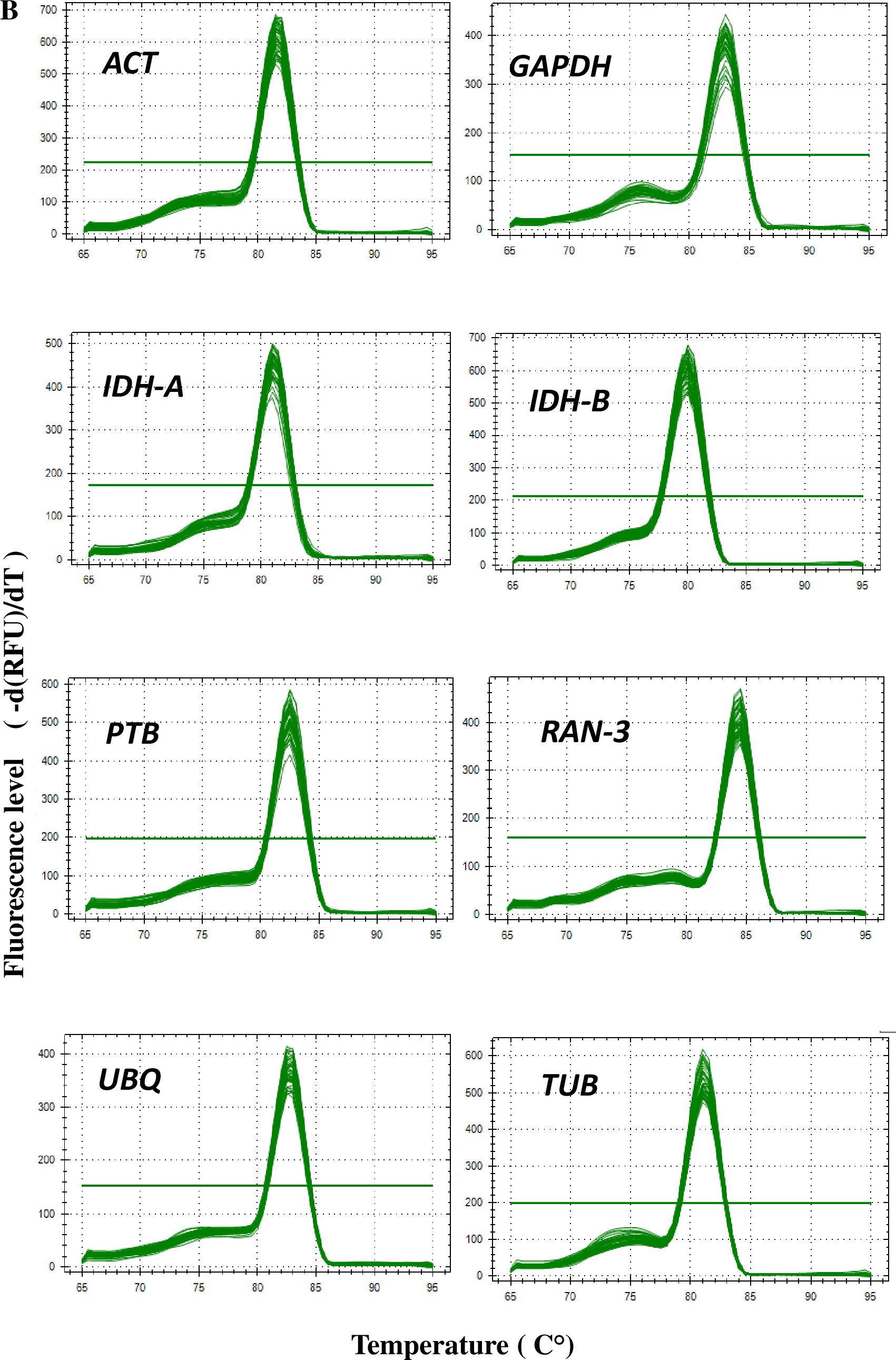

Supplement: S4 Fig — The curves were obtained from three technical replicates of three biological replicates (21 diurnal/circadian samples) for (A) CHEN-109 and (B) Titicaca. (TIF) [file pone.0233821.s007.tif]

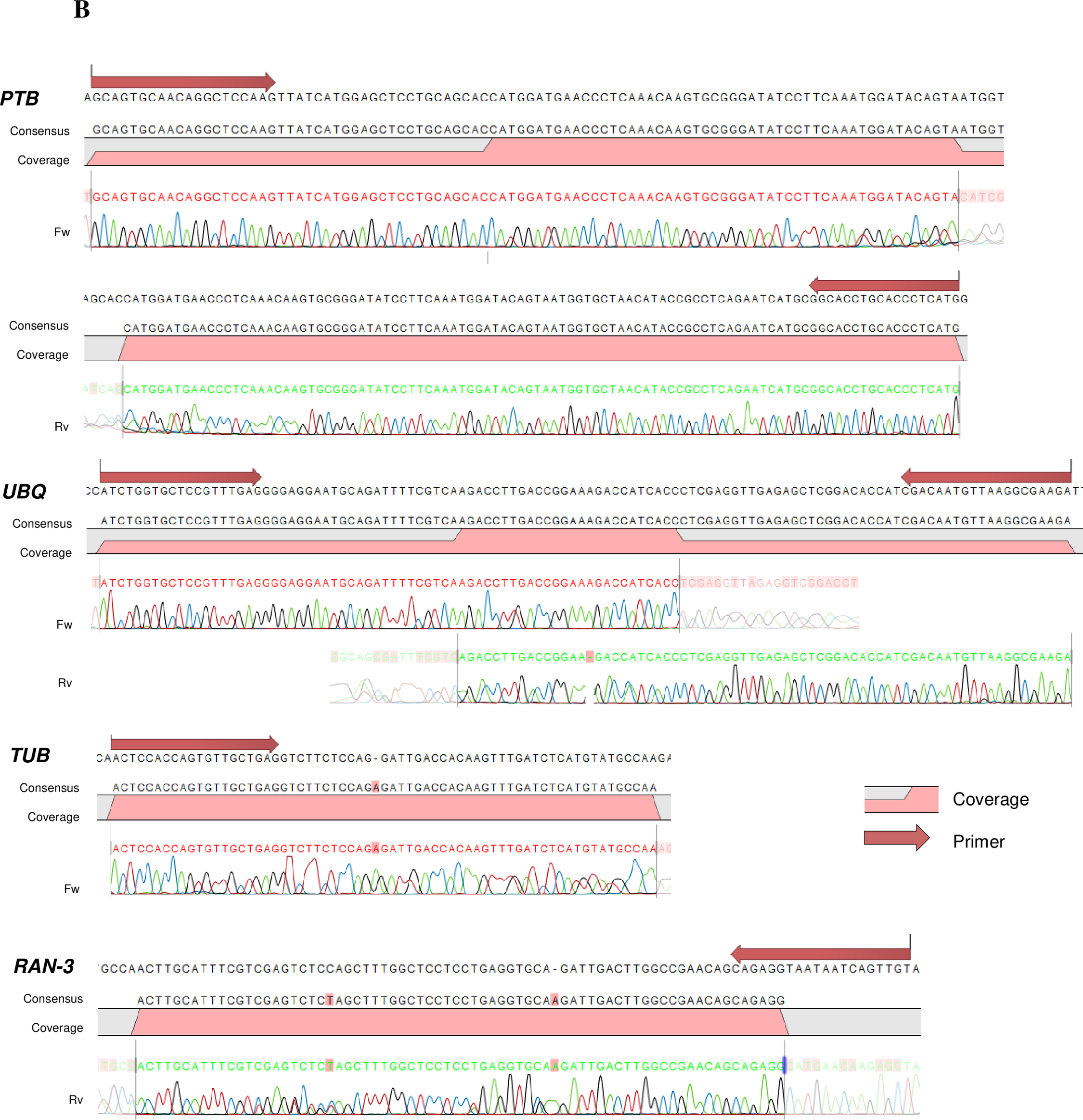

Supplement: S5 Fig — Sequencing results of (A) ACT, IDH-A, IDH-B (B) PTB, UBQ, TUB and RAN-3. Sequences correspond to Titicaca accession aligned to the reference sequence (accession PI 614886) (Jarvis et al., 2017). Primers are annotated and coverage is shown (captions on the right side). Fw = sequenced with the forward primer, Rv = sequenced with the reverse primer. (TIF) [file pone.0233821.s008.tif]

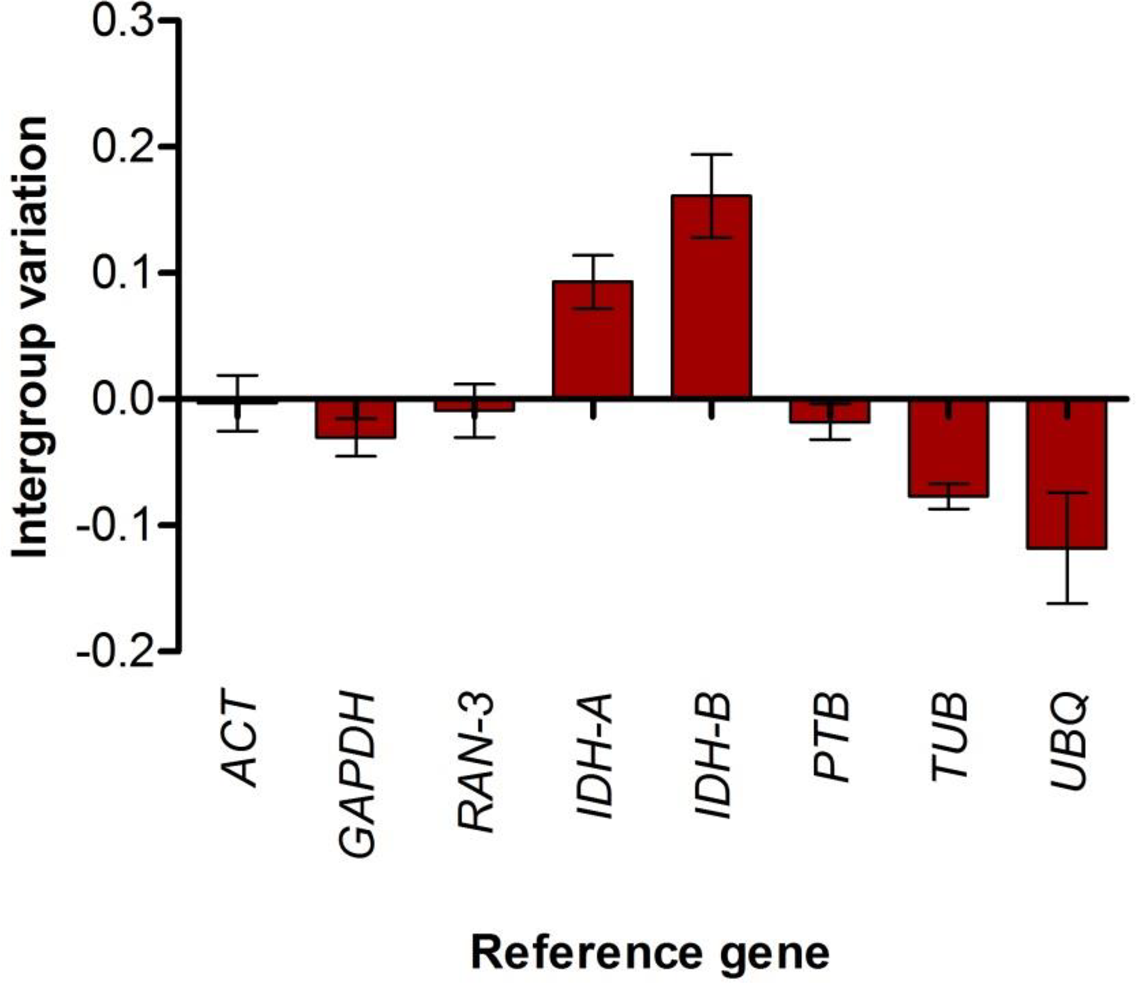

Supplement: S6 Fig — Variation was determined by NormFinder. Error bars represent the intragroup variation. (TIF) [file pone.0233821.s009.tif]

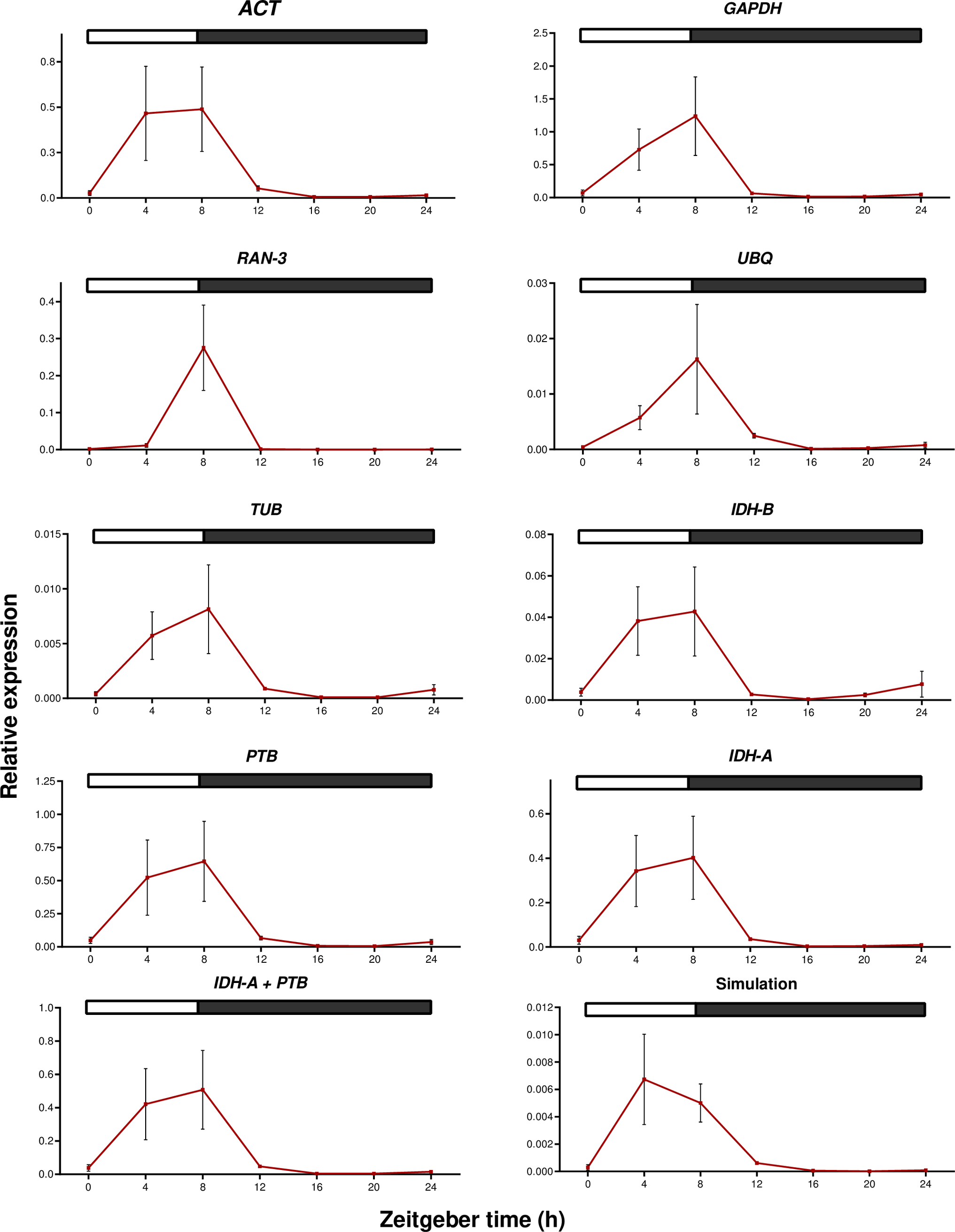

Supplement: S7 Fig — Relative CqGI expression normalized against each candidate gene, the combination of the two best genes for normalization of Titicaca determined by BestKeeper (IDH-A + PTB) and a constant Cq value of 20 (Simulation) are shown. Expression corresponds to the accession PI-587173. The bar at the top indicates light (empty box) and dark (filled box) phases. Error bars represent the SEM of three biological replicates. (TIF) [file pone.0233821.s010.tif]

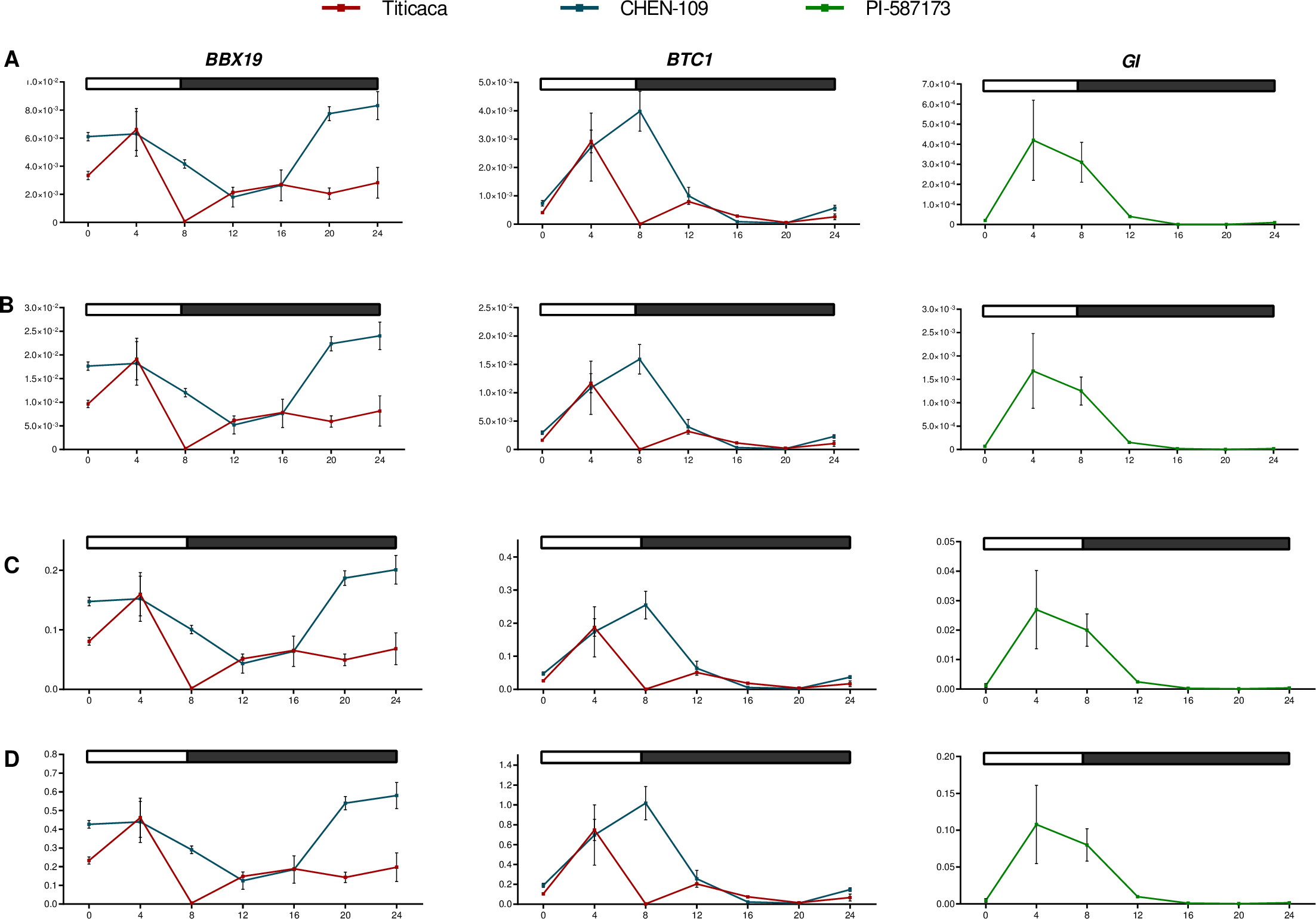

Supplement: S8 Fig — Target genes expression was normalized against a constant Cq value of (A) 16 (B) 18 (C) 22 (D) 24. (TIF) [file pone.0233821.s011.tif]

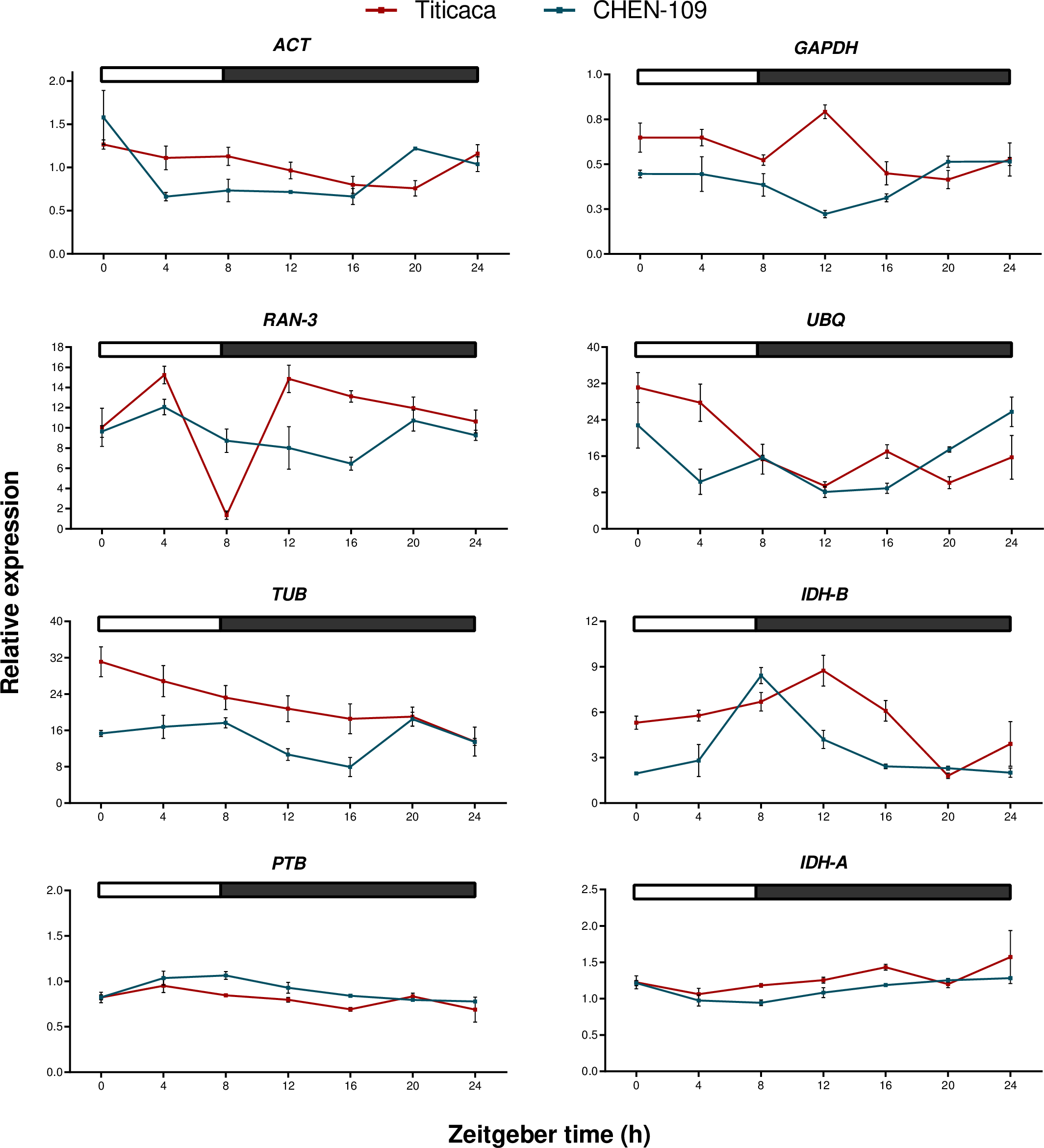

Supplement: S9 Fig — Relative expression normalized against IDH-A and PTB geometric mean. The bar at the top indicates light (empty box) and dark (filled box) phases. Error bars represent the SEM of three biological replicates. (TIF) [file pone.0233821.s012.tif]

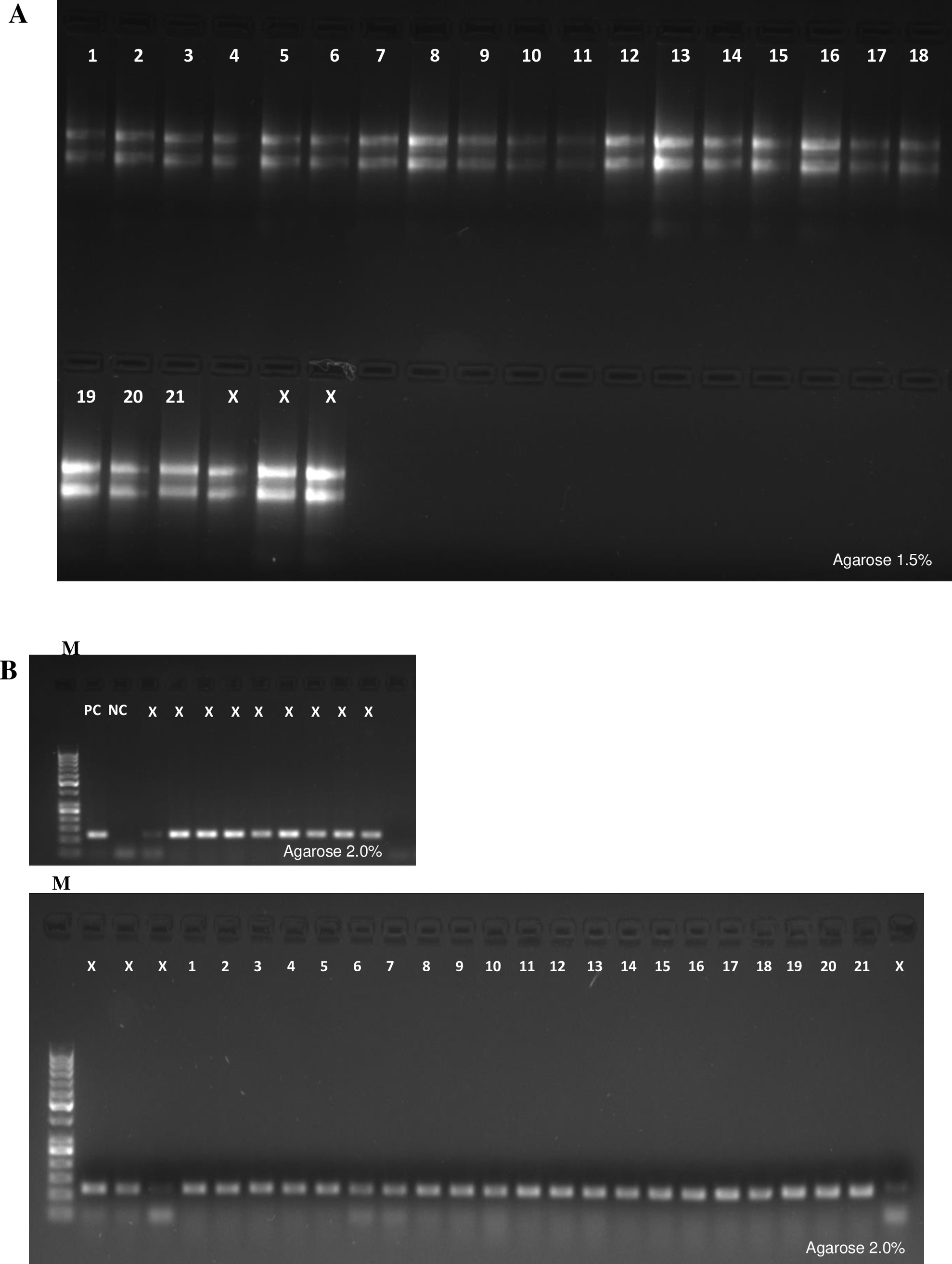

Supplement: S1 Raw images — Full-length gels corresponding to cropped gels in (A) S1A Fig and (B) S1B Fig. Lanes labeled with an “X” in (B) correspond to different primer combinations tests for cDNA amplification by standard PCR. M: 50 bp ladder. (TIF) [file pone.0233821.s013.tif]
