# Supplementary material for: Validation of suitable genes for normalization of diurnal gene expression studies in Chenopodium quinoa
Source: PLoS One. 2021 Mar 11;16(3):e0233821. doi: 10.1371/journal.pone.0233821 (PMC7951847; doi:10.1371/journal.pone.0233821)
Supplement: Attachment — Submitted filename: Response to reviewers_10.02.20.docx [file pone.0233821.s015.docx]

**Response to reviewers**

Reviewer #1: I would like to thanks the authors for answer my concerns and clarify most of my points. However still I think that represent more a methodology rather than address a biological question. Based on that I will leave the decision to accept/reject the manuscript in the hands of the Editor.

*Answer:*

We thank the reviewer for spending the time to review the manuscript again. We feel like we have addressed this point in the last revision under Introduction lines 31 to 35 and 61 to 64.

Reviewer #2: The authors of the manuscript "Validation of suitable genes for normalization of diurnal gene expression studies in Chenopodium quinoa" improved their article substantially in the course of revision and clarified most issues.
However, one of my concerns persists. It is the simulation method, when the authors took the constant Cp value 20 across all the samples. This approach would work supposing that exactly identical amount of cDNA was added to each qPCR reaction. The authors relied on Nanodrop measurements of RNAs before performing reversion transcription and DNAse treatment. However, DNAse treatment and reverse transcription may generate tube-to-tube differences in the final cDNA yield. Thus, I do not consider this simulation to be an appropriate tool to verify reference gene stability. Fluorescence-based estimation of cDNA amount shall be used instead to measure equality of input to RT PCR reaction. Please, consult the paper Libus et al., Biotechniques 2006.

*Answer:*

We thank the reviewer for their comments. We agree that fluorescent methods may be better suited to quantify RNA or to estimate the amount of cDNA, especially when different amounts of RNA and/or contaminants can be expected, for instance because different tissues were used, or strong stresses were imposed on the plants. In this study, we focused on one tissue only (leaves) and plants were all at the same developmental stage. Moreover, the Nanodrop-spectra suggest that samples are similar in purity. Nevertheless, we have discussed the drawbacks in lines 396-404 and 469-471. We have also cited the mentioned reference as an advisable method for quantification of cDNA (line 404). The use of the simulation is intended to work together with the widely used methods to, mainly, disregard the reference genes that produce a highly distorted pattern from the “expected” one (already mentioned in line 389). We have followed the MIQE guidelines, which allows the use of Nanodrop (added S3 Table and line 596).
